# Hair Cortisol Concentrations in Feral Horses and the Influence of Physiological and Social Factors

**DOI:** 10.3390/ani13132133

**Published:** 2023-06-27

**Authors:** Sarah A. Medill, David M. Janz, Philip D. McLoughlin

**Affiliations:** 1 Department of Biology, University of Saskatchewan, Saskatoon, SK S7N 5C8, Canada; 2 Department of Veterinary Biomedical Sciences, University of Saskatchewan, Saskatoon, SK S7N 5B4, Canada; david.janz@usask.ca

**Keywords:** cortisol, hair cortisol concentration, noninvasive sampling, feral horse, *Equus ferus caballus*, reproductive costs, sociality, resource limitations

## Abstract

**Simple Summary:**

Hair tissue records a longer activity profile of an animal’s hypothalamic–pituitary–adrenal axis, and resulting production of cortisol, compared to other matrices (e.g., blood serum, saliva, or feces). Cortisol is a key hormone involved in mobilizing stored energy reserves to assist in meeting physiological demands, but production can also be triggered by psychological stimuli or perceived demands. For horses (*Equus ferus caballus*), most studies investigating hair cortisol concentrations (HCC) have used domesticated individuals where nutritional requirements are fully met, social structures may not be as dynamic, or reproductive behaviors and physiological demands are inhibited. Here, we investigated HCC from 282 samples of hair collected from a population of feral horses that exist under fully natural conditions, including a self-determined social structure, at Sable Island, Nova Scotia, Canada. We found that HCC was predominantly associated with sex, age, body condition, and year effects but, also, for females, the presence or absence of a foal. Female HCC was further influenced by social factors, including harem size and the number of adult males not associated with a band (i.e., bachelors) that coexisted in their home range. In addition, we evaluated biological (e.g., hair color) and procedural factors (sample mass and hair texture) against observed HCC.

**Abstract:**

Cortisol is a glucocorticoid hormone produced during activation of the hypothalamic–pituitary–adrenal axis (HPA) in response to psychological or physiological demands. High amounts of circulating cortisol can be found in individuals experiencing energetically demanding physiological events, such as pregnancy, lactation, injury, or starvation, but, also, in individuals who may have less obvious HPA activation from social situations. The feral horse population on Sable Island (Nova Scotia, Canada) provides an opportunity to look at hair cortisol concentration (HCC) as a proxy for circulating cortisol concentration to better understand physiological correlates. The horse’s complex social structure also allows us to look at how the population and group structure may influence HPA activation. Hair samples (*n* = 282) were analyzed from 113 females and 135 males. Females with dependent offspring (foals) had higher HCC than those females without dependent offspring (*p* = 0.005). Horses in poor body condition were also more likely to have higher HCC (females: *p* < 0.001, males: *p* = 0.028); females had greater variation in the body condition index (BCI), which also correlated with foal production. In general, the top-ranked models describing female cortisol levels included age, BCI, presence of a foal, as well as social measures such as harem size and the number of bachelors in the vicinity. The top model describing male cortisol levels included age, BCI, and year of collection only, and the number of bachelors in the home range appeared in subsequent, though still high-ranked, models. Among the variables not of direct interest, we found some significant results relating to hair color and hair texture. Differences in HCC patterns between feral and domestically kept horses (e.g., age and sex) are likely linked to periods of resource limitations, particularly for individuals experiencing energetically demanding processes such as reproduction, illness/parasitism, or related to experiencing the full range of social and reproductive behaviors.

## 1. Introduction

Activation of the hypothalamic–pituitary–adrenal (HPA) axis is known to occur in response to both internal physiological regulations, as well as in response to influences from the external environment [1,2]. Primarily, it is intended to prepare the body for increased energy demands by activating the release of glucose from reserves while diverting resources from other physiological processes such as growth, reproduction, and immunity [1,3]. In the last two decades, wildlife biologists have been interested in understanding the relationships between glucocorticoid concentrations and how that may infer knowledge on how an organism is coping with changes in its own physiology, its habitat, resources, or social environment [2,4,5,6,7,8].

The merits and disadvantages of the various matrices used to determine glucocorticoid concentrations have been discussed thoroughly by others [9,10,11,12]. Blood serum, saliva, urine, feces, hair, and claws/fingernails all differ in several properties, but most importantly, they vary in the period of time represented by the hormones recovered. Blood, saliva, urine, and feces reflect very recent circulating levels and are frequently affected by circadian or other stochastic events, while slow-growing matrices such as hair, claws, or fingernails represent glucocorticoid accumulation over longer periods of time. Hair is an attractive matrix, as it is minimally invasive to collect, robust to various storage conditions and exposure [8,13,14], and it offers a prolonged retrospective view of an individual’s HPA axis activity [10,11,15,16,17]. Hair hormone analysis has been used to look at differences between the sexes [18,19,20], different ages or development periods [8,21,22,23], access to resources or energy requirements [24,25,26,27], the reproductive state [24,28], or physical injury [8,14,16,29]. Glucocorticoid levels have also been linked to social correlates such as changes in social group membership [16,30] and aggression given or received in relation to a hierarchal rank [16,31,32,33]. Additionally, HCC has also been used to infer effects of anthropogenic activities on wildlife populations [20,34,35,36,37]. 

While many candidate mammal species may serve as models to understand the relative influence of physiological and social factors on cortisol production, horses (*Equus ferus caballus*) present a superior model for research given our deep understanding of both equine biology and sociality. There is a growing body of work originating from domestically kept horses on equine HPA responses to various physiological and social stressors using HCC, including husbandry [38,39,40,41], injury and illness [40,42,43], strenuous exercise [41,44], novel experiences [45], and seasonality [26,38]. The reproductive state of domestically kept female horses has shown both negligible correlation to cortisol levels [46] and also peak releases of cortisol in late pregnancy [47] and prepartum [48,49]. There is also increased cortisol production among stallions, which are used for breeding [47]. Manipulated domestic groups of horses have shown increased HPA axis activity when faced with new individuals or losing access to familiar associations [50,51,52]. However, there is a limited understanding of how cortisol responses may relate to physiological and social factors in feral horses that experience a natural range of social and reproductive behaviors, resource limitations, and other factors that are often excluded in a domestic setting. Early work used serum cortisol concentrations to evaluate for capture stress [42,53,54], while more recent studies looked at fecal glucocorticoid levels in relation to changes in band memberships or associations [30,55]. Little is known of the relative influence of how physiology and socioecology might interact to govern glucocorticoid production in feral horses. 

The long-term, individual-based study of feral horses on Sable Island, Nova Scotia, Canada, presents an opportunity to explore relative HPA pathway activation under natural conditions in horses. The free-living population (N~500 horses) is entirely unmanaged and has been free from all human intervention since the 1960s (the population was introduced to Sable Island in the mid-1700s). Since 2008, the population has been the subject of a detailed study of population ecology whereby, during the breeding season, we document intrinsic variables (age, sex, body condition, and reproductive state), while socioecological factors such as local density, range conditions, the local sex ratio, social group size, no. of stallions in a band, etc. are also assignable to each horse [56,57,58,59,60]. This population is also an excellent model to test the balance of intrinsic vs. extrinsic factors on glucocorticoid regulation as, while the horses exist in entirely natural conditions, they are the only grazing mammal (no competing herbivores), nor are there predators that may have an influence on an individual’s exposure to stressful events. Using hair collected from known individuals as our recording matrix, we examine how glucocorticoid hormone accumulation relates to individual-specific influences of physiological and social situations. 

Our work here is largely exploratory, i.e., determining what correlates can be drawn between physiological and sociological states and the observed hair cortisol concentration (HCC). We expected to see differences in the HCC patterns compared to those observed in domestically kept horses. We predicted that female Sable Island horses would have higher HCC when producing foals, and horses in poor body condition, perhaps not obtaining adequate resources or experiencing underlying illness, injuries, or parasite loads preventing them from acquiring sufficient resources, would also likely have higher HCC. Among male horses, social position may also influence energetic requirements based on the different responsibilities between stallions (dominant males, which control a harem of females) and their bachelor, or non-harem-holding, counterparts. As in domestic horses, and one other population of feral horses [30], we anticipate that changes in social associations could lead to greater HCC. 

We examined HCC relationships using linear mixed effect models, including the strongest physiological (sex, age, body condition index, and reproductive/social state) and phenotype (e.g., hair color) with sociological correlates that included both local population demographics (horse density, number of bands, or bachelors in an individual’s home range), as well as variables characterizing an individual’s immediate social situation (harem or band size). Models were built separately for males and females to investigate how the sexes may respond differently to various stressors in their environment. We used generalized linear models to investigate binary data (e.g., disperse/remain in group, foal presence/absence). Additionally, we considered variables that may be present but not of interest, such as hair color and texture, and procedural influences when using samples with a range of original weights.

## 2. Materials and Methods

### 2.1. Study Area and Species

**Figure 1:**
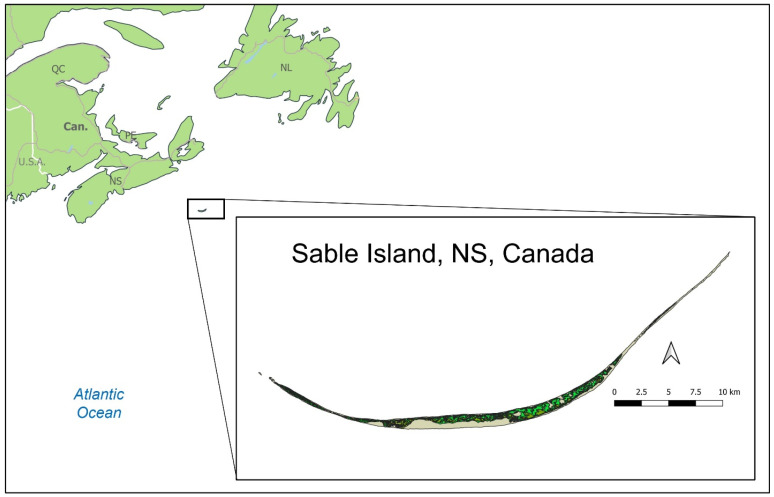
Location and vegetation distribution of Sable Island, Nova Scotia (NS), Canada. A population of approximately 500 feral horses range freely on the island. Vegetated surfaces are represented by light and dark green.

Sable Island is located 275 km east of Halifax, NS, Canada, in the Atlantic Ocean (43.933021° N, −60.005515° W). It is a vegetated sandbar that is approximately 38 km long and has a maximum breadth of 1.3 km, tapering to form a linear crescent shape (Figure 1). Vegetation on the island is predominantly marram (*Ammophila breviligulata*) grassland and heath, all growing on a substrate composed entirely of sand and what little organic material remains from vegetation or animal life. Being the only terrestrial mammal on the island, there are no other competitors nor predators influencing the horse population. As there is no supplemental feeding of the horses, their population is self-regulated based on available resources, climate, or other internal factors [56,61,62]. 

The population of feral horses has existed on the island since the mid-1700s and has long established a wild-type social structure of harem defense polygyny. The horses roam uninhibited throughout the island, apart from a few areas around structures where they are excluded by fences. There have been no management or interventions within the population since the early 1960s when they received protection from harassment or removal through the Canadian Shipping Act (2001). Management of the island and the wildlife therein became the responsibility of Parks Canada in 2013, and the horses are considered a naturalized species and protected as wildlife under the Canada National Parks Act (2000). 

### 2.2. Annual Survey

Each summer since 2008, the horses have been surveyed and data on their location, associations, reproductive status, and health collected several times over the July–August field season [63]. Year of birth is determined for horses seen as foals or yearlings; we have known ages for individuals born since the start of the project in 2008, including those born in 2007 (seen as yearlings in 2008). Photos taken from a reconnaissance visit in 2007 allowed age assignment to a portion of the 2006 cohort. This results in us having known-aged individuals up to 6 years old and a group of unknown-aged individuals that may include horses 6 years old and older at the time samples were collected. Individual horses are readily identifiable using their natural markings, whorl patterns, scars, and other characteristics. Identifications are done post-survey using photographs linked with field data and samples and compared to the previous year’s photos in the database [59,63].

Female reproductive status is determined by the presence or absence of a foal during the summer survey or a yearling present in the subsequent year’s survey (i.e., parturition occurred after the previous survey ended). Male social positions are identified based on behavior [64,65,66], with individuals classed into one of four groups during sampling as being a dominant band stallion (Stallion), a subordinate band stallion (Tag), an adult male ≥ 5 years old unassociated with reproductive-aged females (Bachelor), or a male (3 or 4 years old) that may still be with its natal band or dispersed and living as a bachelor (Immature). Male horses may produce semen as young as two years old [67]; however, males that are less than 5 years old living in a natural social structure are rarely seen consorting with females with the intention to reproduce [65,68]. For bands, the total band size (a count of all individuals, including foals), as well as harem size (number of females ≥ 2 years old), was determined.

### 2.3. Body Condition Index

The body condition index (BCI) was determined by evaluating photographs taken during the field survey. Individuals would be graded on a scale of 0–5, with half-point scoring (zero being emaciated and 5 being very obese). Three body regions on an individual were rated: hips, rib cover, and backbone, and half points were used when a difference was found between the three regions. This scale follows the criteria set out in Carroll and Huntington [69]. 

### 2.4. Location and Home Range Establishment

A median (centroid) location along the length of Sable Island (Universal Transverse Mercator or UTM-X and -Y) for an individual is determined using all the observations of that horse during the survey year. From these median locations, a buffer of 8000 m captures the 99th percentile of movements of all horses along the length of Sable Island [56]. These buffers represent the maximal home range, or area used, and describe individual-specific exposure to environmental and social variables. For example, within these buffers, we calculated a measure of the horse density (no. of horses per vegetated km^2^), the number of bands, and the number of bachelor males which median locations also fall within the defined radius. The location variable used in this research is the UTM-X (longitude) coordinate, which reflects the position along the east–west orientation of the island (Figure 1).

### 2.5. Sample Collection and Processing

In 2011 and 2012, tail hairs were collected for microsatellite DNA analyses and for hormone analysis (this study) under our institutional animal care permit (University of Saskatchewan UCACS #20090032). The collection methods included collecting samples that were observed to be scratched off onto previously cleaned natural and artificial hair snags (Velcro strips set out on known rub structures); however, most samples were collected by approaching and directly pulling the hairs from unrestrained individuals. Hair collected in the field was handled with nitrile gloves, placed in labeled envelopes, and stored under ambient temperatures in a dark location. 

Only hairs with the root attached were used in this analysis to ensure the most recent hair growth was used. We removed 4 mm of the root end to retain for DNA analysis and used the next 5 cm segment to evaluate the cortisol concentration. Tail growth rates of domestic horses have been reported as between 0.066 and 0.081 cm per day [70,71,72,73,74,75]. Based on these rates of growth, the 5 cm segment would represent 67–82 days prior to sample collection, excluding the most recent 5–6 days associated with the 4 mm portion of hair root that was removed. Hair samples were collected during roughly the same time period in both years: between 24 July and 23 August in 2011 (*n* = 50) and 20 July to 17 August in 2012 (*n* = 232). Peak parturition in the Sable Island horse population occurs in May, and most breeding occurs during May–July [76,77]; therefore, the cortisol profile within the hair shaft is likely to coincide with this period of high energy demands.

### 2.6. Laboratory Analysis

We analyzed hair samples following the procedures described by Macbeth et al. [13], with the following modifications relating to the use of hair segments. Methanol washes used to remove exogenous sources of hormones and contaminants (e.g., from sweat or feces) were performed after the removal of the 4 mm root end but before the 5 cm segment was isolated from the full hair shaft. The focal segment of hair was further cut into smaller pieces and ground into a powder using a Retsch MM 301 Mixer Mill (Retsch Inc., Newtown, PA, USA). Up to 25 mg of powdered hair sample was analyzed by extracting hormones with HPLC grade methanol over 24 h on a slow rotator, centrifuged, and the supernatant collected. Samples were rinsed with additional HPLC grade methanol, vortexed, centrifuged and the supernatant collected again. This was repeated until a total of three collection and rinse cycles occurred. The pooled supernatant was dried at 38 °C under a gentle nitrogen gas stream. Steroids were then concentrated at the bottom of the test tube by consecutive methanol washes of decreasing volume (0.4, 0.2, and 0.15 mL) and drying as before. Samples were reconstituted using 0.2 mL phosphate buffer provided in the EIA kit (left 12 h at 4 °C in the dark). The cortisol concentrations were quantified using commercially available enzyme-linked immunosorbent assay kits (EA-65 Cortisol EIA kit, Oxford Biomedical, Oxford, MI, USA) validated for use on equine hair. The intra-assay and inter-assay percent coefficients of variation (%CV) for the cortisol EIA were 6.8% (*n* = 6) and 8.3% (*n* = 12), respectively. The limit of detection for cortisol was 0.02 ng/mL, and strong parallelism between the kit standard curve and serially diluted equine hair extracts was observed (SPSS curve estimation; R^2^ = 0.997, *p* < 0.001).

### 2.7. Data Analysis

All statistical analyses were performed using R version 4.2.2. [78]. Data were evaluated for normal distribution (Shapiro–Wilk test) and outliers (R package: outliers [79]). For simple one- or two-factor comparisons, we used linear mixed effect models (LME; packages: lme4 [80] and lmerTest [81]), which allowed us to include horse IDs as a random variable to account for individuals with repeated measures across years (17 males and 17 females) and, in some cases, when we wanted to account for variations in HCC related to hair color, age, or year. When looking at binary response variables, we used generalized linear mixed effect models (GLME). Analyses and figures were created using the following packages: lme4 [80], tidyverse [82], lmerTest [81], pROC [83], and emmeans [84]. The model type, LME or GLME, and which variables were considered as fixed or random, are presented alongside the results. In a few of the analyses, we were unable to include the horse ID as a random factor or it would lead to overfit models due to the uneven distribution of data among some levels of the factors. We identified where this occurs.

Factors potentially affecting HCC from a procedural point of view may be related to the initial mass of hair used for the extraction or hair texture. When possible, we used a maximum of 25 mg of powdered hair, but for 72% of individuals, there was insufficient hair collected to reach that limit (range = 2.2–25 mg powdered hair). Hair texture was a measure of coarseness and calculated as the total mass of 5 cm segments (before grinding) divided by the number of hairs in the sample to give us a mg·hair strand^−1^ measure. We compared observed HCC to the coarseness and ground sample mass using linear mixed effect models to account for repeated sampling of some individuals (random factor: horse ID). Similarly, we also evaluated HCC for differences between sex, age, year, ordinal day, median location on the island (UTM-X), and hair color among males and females combined. After evaluating HCC responses to variables at the population level, we built models separately for males and females to focus on factors specific to them. For females, this included foal or yearling presence/absence, BCI, harem or band size, and dispersal. For males, we looked at their social position (Immature male, Bachelor, Stallion, or Tag), along with body condition, harem or band size, and dispersal. 

Last, for both males and females, we built inclusive models that were used to evaluate the relative importance of biological and social variables. We first built a model focusing on biological variables (BCI, foal, age, year, and location on the island) and their 2-way interaction terms and used AIC_c_ model selection [85] (Equations (S1) and (S3)). We investigated our data relating to the social landscape within an individual’s home range (e.g., adult sex ratio, number of bachelors, number of bands, horse density, harem size, and band size) for collinearity (Figures S1 and S2) and removed variables that were strongly correlated with other explanatory variables [86]. Model development and evaluation steps were taken to investigate HCC correlations to the remaining social or home range-level variables (Equations (S2) and (S4)). The above results are presented in the Supplemental Materials. The factors that appeared in the top-ranked models for both biological and social influences on HCC were then combined to create models that best explained the observed variations in HCC among females (Equation (1)) and males (Equation (2); packages: lme4 [80] and MuMIn [87]).

## 3. Results 

### 3.1. Initial Data Exploration

A total of 359 samples of tail hair were collected from Sable Island horses and analyzed to determine the cortisol concentration. Thirty-nine of these samples returned values that were below the detection limit; the ground mass of these samples ranged from 2.22 to 13.45 mg, mean = 4.6 mg). Twelve more samples had coefficients of variation that exceeded 15% and were excluded from further analysis. Grubb’s tests for outliers identified three samples as outliers, and these were excluded. Four additional sample results were removed, because they were from ages that were poorly represented in the rest of the dataset (one sample from an individual aged 0, one sample for age 1, and two samples for age 2 (one male, one female); we removed duplicate samples that were taken from an individual in the same year (*n* = 19) by retaining the results with the lowest %CV, which suggests a more precise assay result. Samples taken in different years for the same individual were retained (*n* = 34). The final number of samples used in the following analyses was 282; the breakdown was 130 samples from female horses (113 individuals) and 152 from male horses (135 individuals). Fifty of the samples were collected in 2011, and the remaining 232 samples were collected in 2012. The distribution of HCC in pg·mg^−1^ was skewed to the right (Shapiro–Wilk test, W = 0.809, *p* < 0.001), but log transformation produced a normal distribution for statistical analysis (W = 0.994, *p* = 0.339).

### 3.2. Procedural Considerations

**Figure 2:**
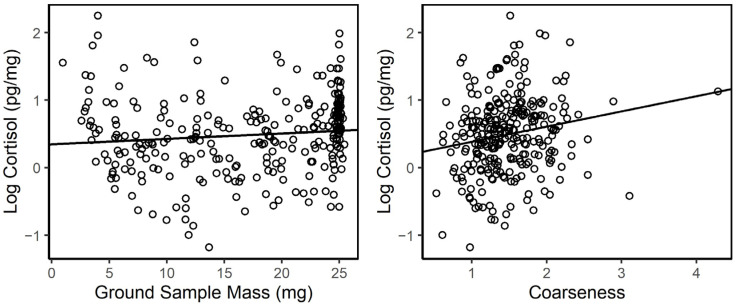
Hair cortisol concentration showed an insignificant increase with the increase in the ground sample mass used for extraction (left, R^2^ = 0.11, *p* = 0.059). Hair coarseness, a measure of the sample hair weight divided by the number of hairs, had a weak but significant relationship with the hair cortisol concentration (right, R^2^ = 0.18, *p* = 0.002).

The procedural considerations we investigated included the weight of the ground sample that underwent extraction and the coarseness of the hair. We had very little control over the quantity of hair we could collect either from rubs or opportunistically, so it was of interest to know how small of a sample from which we could successfully extract cortisol. Successful values for cortisol (i.e., did not return a reading below the detection limit) were obtained from samples with a ground mass as low as 0.97 mg or from as few as three hairs. The correlation between the ground sample mass and the observed log cortisol was not significant (R^2^ = 0.11, *p* = 0.059), and the linear mixed effects model (LME; fixed: sample mass, random: horse ID; *p* = 0.083) supported a nonsignificant influence of the ground sample mass on the HCC (Figure 2). The observed coarseness of the hair (sample mass divided by number of hairs in the sample) showed a weak but significant correlation with the HCC (R^2^ = 0.18, *p* = 0.002) that persisted even after investigating and removing data points with high leverage in the model. Statistical testing accounting for the repeated samples for 34 individuals was also significant (LME; fixed: coarseness, random: horse ID; *p* = 0.002); however, the pattern of HCC to hair coarseness was quite scattered, and the low coefficient suggested that only a very small fraction of the variation in HCC was explained by hair coarseness.

### 3.3. Biological Considerations

#### 3.3.1. Sex and Age

**Figure 3:**
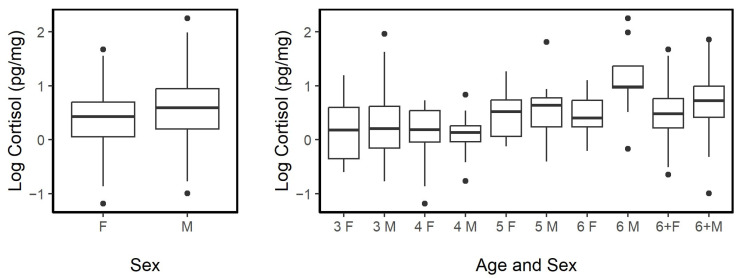
Hair cortisol concentrations (HCC) of female (F; *n* = 130) and male (M; *n* = 152) feral horses were overall significantly different (*p* = 0.004). Feral horses aged 6 and those identified as adults (6 + years of age) had higher HCC than younger individuals. Sample sizes as follows: 3-year-old females (3 F, *n* = 25) and 3-year-old males (3 M, *n* = 30); 4 F, *n* = 15; 4 M, *n* =15; 5 F, *n* = 9; 5 M, *n* = 11; 6 F, *n* = 9; 6 M, *n* = 9; 6 + F, *n* = 69; 6 + M, *n* = 87. The median is represented by a black line, the box represents the interquartile range (IQR), and the whiskers are 1.5 × IQR.

Overall, males had a mean ± sd HCC of 2.17 ± 1.46 pg·mg^−1^ (*n* = 152, range = 0.37– 9.50 pg·mg^−1^), while the female mean was 1.64 ± 0.81 pg·mg^−1^ (*n* = 130, range = 0.31–5.33 pg·mg^−1^). The difference in HCC between the males and females was significant (LME, fixed: sex, random: horse ID; *p* = 0.003; Figure 3); however, this may have been driven mostly by the significant interaction between sex and individuals which were 6 years old; males had significantly higher cortisol at that age than females (LME, fixed: sex × age, random: horse ID, *p* = 0.028). Overall, the HCC tended to be higher in both male and female adults aged 6+ than in younger individuals (LME, fixed: sex × age, random: horse ID; *p* = 0.019; Figure 3). 

#### 3.3.2. Sample Collection Year and Day

For 34 individuals for which there was a hair sample collected in both years (17 females and 17 males), a paired *t*-test of HCC did not show a significant year effect (*p* = 0.981). A comparison of all the data from both years did, however, show an overall significant difference between years with higher cortisol levels in 2012 (LME; fixed: year, random horse ID; *p* < 0.001). The ordinal day the sample collection occurred was not a significant factor influencing the HCC (LME; fixed: day, random: horse ID; *p* = 0.500).

#### 3.3.3. Median Location

No discernable correlation between the HCC and an individual’s median location on the island could be found (LME, fixed: UTM-X, random: horse ID; *p* = 0.516).

#### 3.3.4. Hair Color

Hair color was also a variable reported capable of introducing potential bias into the hair hormone analysis [14,32,88]. We found that sorrel individuals appeared to have significantly lower cortisol than individuals exhibiting other hair phenotypes (LME; fixed: hair color, random: horse ID; *p* = 0.007); however, there were fewer sorrel (*n* = 11) and flaxen (*n* = 3) individuals than there was chestnut (*n* = 28) or especially black (*n* = 240) amongst our samples. An unequal sample size reduced the power of this test and increased our potential for a type 1 error [89].

### 3.4. Cortisol Levels in Females

#### 3.4.1. Establishing Random Variables

Amongst females, the variable for hair color was statistically significant for a single flaxen individual (LME; fixed: hair color, random: horse ID; *p*_flaxen_ = 0.011). We decided to remove this individual to simplify the models and to not account for hair color as a random factor for the remaining female samples. There were 24 samples from females collected in 2011 and 105 samples collected from females in 2012. Seventeen of these individuals had samples collected in both years. Depending on the hypothesis being evaluated, horse ID, year, or age (given the differences observed above) may be included as a random effect in the models.

#### 3.4.2. Age and Reproductive Effort

**Figure 4:**
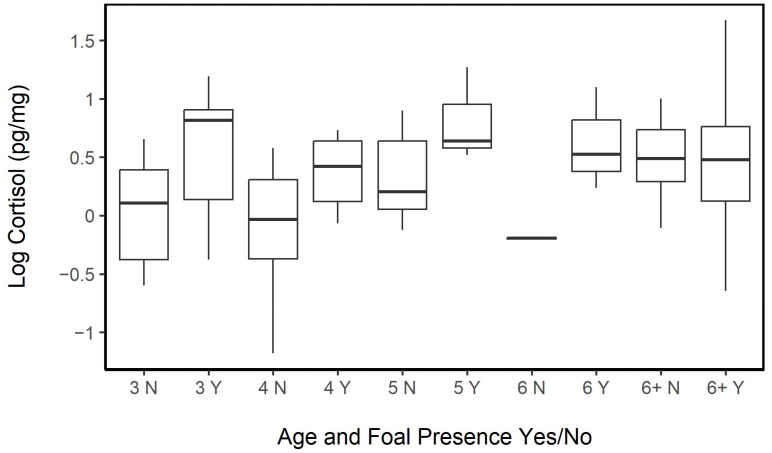
Hair cortisol concentrations in females with (Y) or without (N) a foal were significantly different (LME, fixed: age, foal Y/N, age × foal Y/N, random: Horse ID; *p* = 0.005) and tended to have pronounced differences in younger females, while these differences lessened when looked at in mature individuals (*p* = 0.012). Sample sizes as follows: No foal and 3 years old (3 N, *n* = 16); Foal present and 3 years old (3 Y, *n* = 9); 4 N, *n* = 6; 4 Y, *n* = 11; 5 N, *n* = 6; 5 Y, *n* = 3; 6 N, *n* = 2; 6 Y, *n* = 7; 6+ N, *n* = 21; 6+ Y, *n* = 48. The median was represented by a black line, the box represented the interquartile range (IQR), and the whiskers are 1.5 × IQR.

Females with a foal (*n* = 78, mean ± sd = 1.82 ± 0.88 pg·mg^−1^, range = 0.52–5.33 pg·mg^−1^) tended to have higher hair cortisol levels than females without a foal (*n* = 51, mean ± SD = 1.39 ± 0.60 pg·mg^−1^, range = 0.30–2.73 pg·mg^−1^). The presence or absence of a nursing foal was related to a significant difference in HCC (LME; fixed: age, foal Y/N, age × foal Y/N, random: horse ID; *p* = 0.005). A significant interaction between age and the presence or absence of a foal was also observed (*p* = 0.012). Hair cortisol concentrations in young females tended to be lower in those without a foal compared to same-aged counterparts with offspring, while, among the horses in the age group 6+, the difference in hair cortisol was negligible for those with or without foals (Figure 4). 

**Figure 5:**
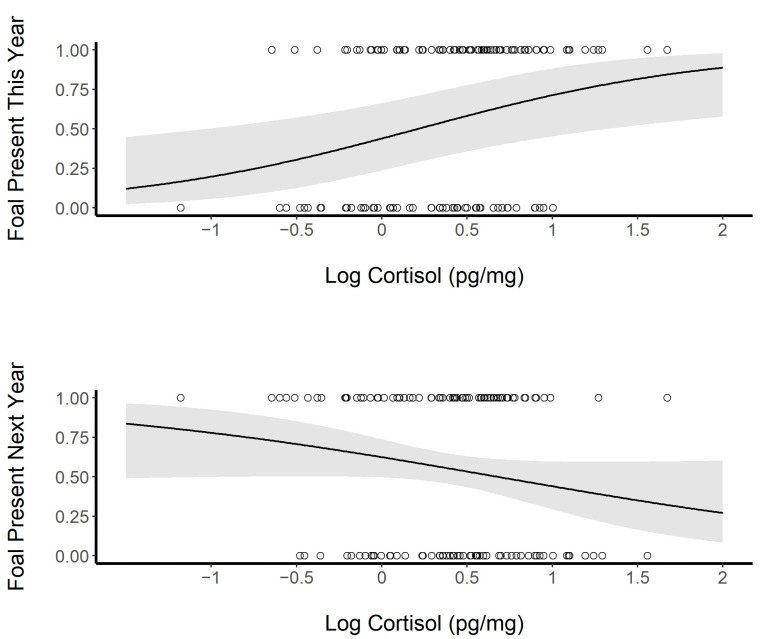
Log cortisol concentrations in the hair of female horses and the presence (1) or absence (0) of a foal in the year of sampling (top; *p* = 0.009), and a comparison of the current season’s hair cortisol concentrations and the presence (1) or absence (0) of a foal in the following year (bottom; *p* = 0.082). The shaded area represents the 95% confidence interval.

We used a GLME to look at foal presence or absence as a function of the observed HCC but were unable to fit horse ID as a random variable for the current year due to model overfitting. However, we have already established a significant relationship between the HCC and foal presence in the LME. The results of the GLME model for the current presence or absence of a foal remained significant even after accounting for the variations present in year and age (GLME; fixed: log HCC, random: year and age; *p* = 0.009; AIC = 168.9, null model AIC = 174.2; Figure 5). However, HCC was not a reliable predictor of the presence or absence of a foal in the subsequent year (GLME; fixed: log HCC, random: horse ID; *p* = 0.084; AIC = 179.5, null model AIC = 180.7). The HCC of the female did not differ based on the sex of the foal present (GLME, fixed: log HCC, random: horse ID; *p* = 0.980; AIC = 113.7, null model AIC_c_ = 111.7). 

**Figure 6:**
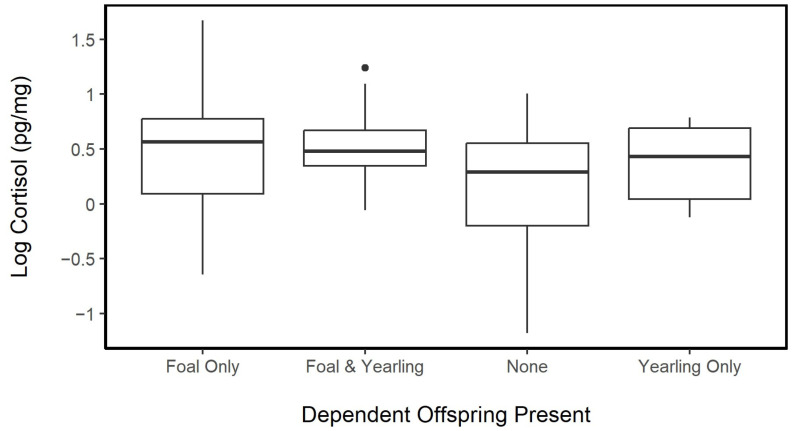
Log hair cortisol concentrations (pg·mg^−^^1^) of females with different reproductive demands. Females with foals only (*n* = 54) and females with foals and yearlings (*n* = 25) had significantly higher log cortisol concentrations than females with no accompanying offspring (*n* = 38, *p* = 0.011). Females with only yearling offspring with them (*n* = 12) were not significantly different from any other classification. The median is represented by a black line, the box represents the interquartile range (IQR), and the whiskers are 1.5 × IQR.

When expanding the focus of the reproductive load to consider females that may also be nursing yearlings or being accompanied by both a yearling and a foal, we found that females with no accompanying offspring had significantly lower hair cortisol concentrations than females that were accompanied by foals (ANOVA, Tukey’s HSD, *p*_adj_ = 0.024) or by foals and yearlings (Tukey’s HSD, *p*_adj_ = 0.024). Females accompanied by yearlings tended to have only slightly higher HCC than females without foals but not significantly (Tukey’s HSD, *p*_adj_ = 0.62). This association was tested using a mixed effect model, and the observed difference was still significant (LME; fixed: reproductive load, random: horse ID, year, and age; *p* = 0.011). Hair cortisol concentrations for females with different reproductive demands from highest to lowest were in the following order: Foal only (*n* = 54, mean ± SD = 1.82 ± 0.98 pg·mg^−^^1^, range: 0.52–5.33 pg·mg^−^^1^) > Foal and Yearling (*n* = 25, mean = 1.79 ± 0.61 pg·mg^−^^1^, range: 0.94–3.46 pg·mg^−^^1^) > Yearling only (*n* = 12, mean = 1.52 ± 0.48 pg·mg^−^^1^, range: 0.88–2.20 pg·mg^−^^1^) > no dependent offspring (*n* = 38, mean = 1.36 ± 0.64 pg·mg^−^^1^, range: 0.31–2.73 pg·mg^−^^1^; Figure 6).

#### 3.4.3. Body Condition

**Figure 7:**
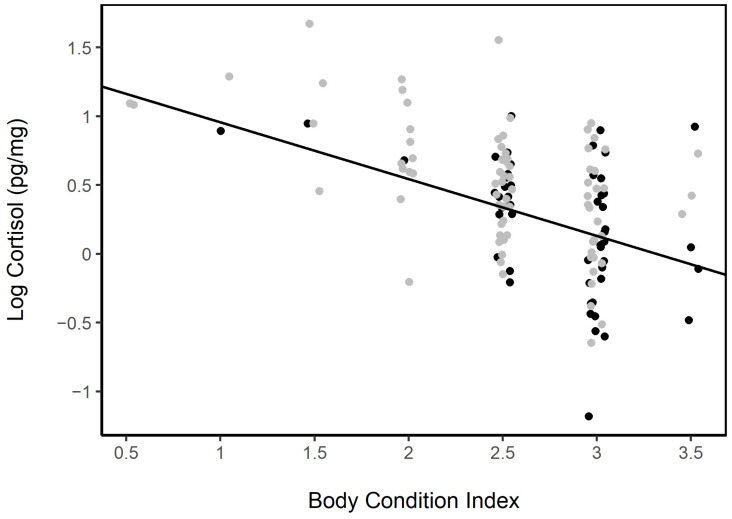
Log hair cortisol concentrations of females with foals (grey) and without foals (black) decreased with increasing the body condition index (0—very poor condition, 5—obese condition; LMER fixed: BC + Foal Y/N, random: age; *p* < 0.001). Females with foals also tended to have lower body condition scores (LME; fixed: foal Y/N, random: horse ID; *p* = 0.004).

The BCI in females was not associated directly to age (LME; fixed: age, random: horse ID; no age group was significant) or year (LME; fixed: year, random: horse ID; *p* = 0.38). The body condition among females on Sable Island was tied to the presence or absence of a foal (LME; fixed: foal Y/N, random: horse ID; *p* = 0.004). The BCI was also a significant variable describing the hair cortisol level (LMER, fixed: BCI, random: age; *p* < 0.001, AIC = 142.1). Including the presence or absence of a foal and the body condition in a model describing HCC improved the model fit (LMER fixed: BC + Foal Y/N, rando: age; *p* < 0.001, AIC = 140.5; Figure 7); however, the interaction term between the BCI and foal presence/absence was not significant, and its inclusion did not improve the model fit (model comparison: χ^2^ = 0.136, df = 1, *p* = 0.712).

#### 3.4.4. Social Structure and Dispersal

Harem size was not significantly correlated with HCC in females (LME; fixed: harem size, random: horse ID; *p* = 0.130), nor was band size (LME; fixed: band size, random: horse ID; *p* = 0.473). 

Female horses showed a nonsignificant trend where individuals that changed bands over the 11-month period before the collection of the hair sample had higher hair cortisol concentrations (dispersed *n* = 53, remained in the band *n* = 76; LME; fixed: dispersed in the previous year, random: horse ID, age; *p* = 0.223). Comparing the observed hair cortisol concentration to whether an individual was seen in a new band the following year suggested that individuals with lower cortisol during sampling had a higher probability of changing bands, while individuals with high HCC were more likely remain in the social group they were currently in (dispersed *n* = 38, remained in band *n* = 91; LME; fixed: dispersed in the subsequent year, random: horse ID, age; *p* = 0.035). 

#### 3.4.5. Best Model of Biological and Social Variables

Variables linked to biological reasons for variations in cortisol were evaluated for their inclusion in an overall best model for explaining female HCC. Year and age were included as fixed factors, along with body condition, the presence or absence of a foal, and location on the island (Table S1). The two-way interaction terms between these variables were also included; no random factors were included, as it led to model singularity errors. The BCI, age and the interaction term age × BCI were found to be in the resulting top models (Table S1). Year, foal presence, and an interaction term between age and foal presence were in the subsequent top models with ΔAIC_c_ < 2.00. 

Social characteristics, as determined within the 8000 m buffers around an individual’s median location, were compared to the cortisol levels to find which sociological or population level experiences explained the most variation in HCC (Table S2). The parameters included the number of bachelors, number of bands, and the density of horses in the vegetated area within the 8000 m buffer around the focal individual’s median centroid. The harem size and overall band size that the individual occupied were inherently strongly correlated (R^2^ = 0.84, *p* < 0.001), so the variable harem size was retained, as it was the stronger correlate to HCC. The median location was also strongly correlated with the other variables, such as bands and density within the 8000 m buffer (Figure S1), so it was also removed from the full model (it had low significance as a factor on its own). These variables, along with all two-way interaction terms, were analyzed to determine their influence on the hair cortisol concentrations (Table S2). Bachelor abundance, number of other bands, harem size, and density, as well as the interaction term of harem × bachelor, were found in the top three models (ΔAIC_c_ = 2.14). The variables found in the top three models for biological and sociological variables were then combined to determine the best model to describe HCC based on AIC_c_ model selection [85], starting with the full model below:

(1) $$C_{ijklnpvr}=B_{I}+F_{J}+A_{K}+Y_{L}+M_{N}+G_{P}+D_{V}+H_{R}+{\left(A\times F\right)}_{J*K}+{\left(A\;\times B\right)}_{K*I}+{\left(M\times H\right)}_{N*R}+e_{IJKLNPVR}$$

where

- C—Log10(HCC pg·mg^−^^1^)
- Fixed Factors:
  B—Body Condition Index, numerical 0–5 (I)
  F—Foal with 2 levels (J = Presence or Absence)
  A—Age, categorical with 5 levels (K = 3, 4, 5, 6, and 6+)
  Y—Year, categorical with 2 levels (L = 2011 or 2012)
  M—Bachelors in 8000 m buffer, integer (N)
  G—Bands in 8000 m buffer, integer (P)
  D—Density in 8000 m buffer, integer (V)
  H—Harem size of focal individual, integer (R)
- $e_{IJKLNPVR}$—residual component
- No random variables were included due to model overfitting/singularity errors.

The overall top model for female HCC included age, BCI, and an age × BCI interaction term, as well as the size of the harem in which the female was associated (Table 1). The next model, with the minimal change in AIC_c_ (0.06), included the number of bachelors found in the individual 8000 m buffers. The factors relating to their reproductive state were in the third top model with ΔAIC_c_ = 0.42.

### 3.5. Cortisol Levels in Males

#### 3.5.1. Establishing Random Variables

Amongst males, the variable for hair color was statistically significant for the sorrel-colored manes (*n*_sorrel_ = 7; LME; fixed: hair color, random: horse ID; *p*_sorrel_ = 0.003); these individuals were left in the dataset, and where possible, we accounted for hair color as a random variable in the model development. There were 26 samples from males collected in 2011 and 126 samples collected from males in 2012. Seventeen of these individuals had samples collected in both years. Depending on the hypothesis being evaluated, horse ID, hair color, and year may be included as a fixed or random effect in future models.

#### 3.5.2. Age and Social Position

**Figure 8:**
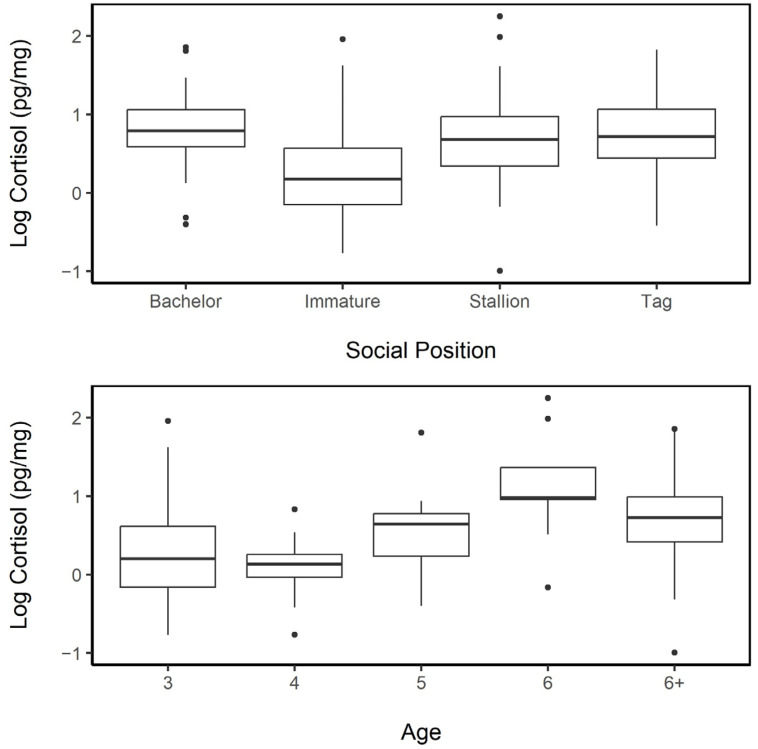
Upper: Log hair cortisol concentrations (HCC) of male feral horses in different social positions (Bachelor *n* = 28, Immature *n* = 43, Stallion *n* = 70, and Tag *n* = 11). Lower: Log HCC of male individuals of known ages of 3 (*n* = 30), 4 (*n* = 15), 5 (*n* = 11), 6 (*n* = 9), and 6+ (*n* = 87). When age is included as a random variable into the models of social position and cortisol concentration, there are no differences between hair cortisol concentrations of males in different social positions. The median is represented by a black line, the box represents the interquartile range (IQR), and the whiskers are 1.5 × IQR.

Hair cortisol concentrations of males occupying different social positions were not found to be different, except for immature males (i.e., individuals aged 3 and 4; Tukey’s HSD: Immature vs. Bachelor *p*_adj_ < 0.001, Immature vs. Stallion *p*_adj_ < 0.001, and Immature vs. Tag *p*_adj_ = 0.05; Figure 8). This likely speaks more to an age effect with some significant difference in cortisol observed among different-aged male individuals. When the model included age as a random factor (along with horse ID, hair color, and year), none of the social positions returned a statistically significant difference (LMER; fixed: male social position, random: horse ID, hair color, year, age; Intercept (Bachelor (*n* = 28)); Immature (*n* = 43, *p* = 0.563); Stallion (*n* = 70, *p* = 0.117); Tag (*n* = 11, *p* = 0.926)), and the model was not significantly different from the null model (χ^2^ = 3.84, df = 3, *p* = 0.279).

#### 3.5.3. Body Condition

**Figure 9:**
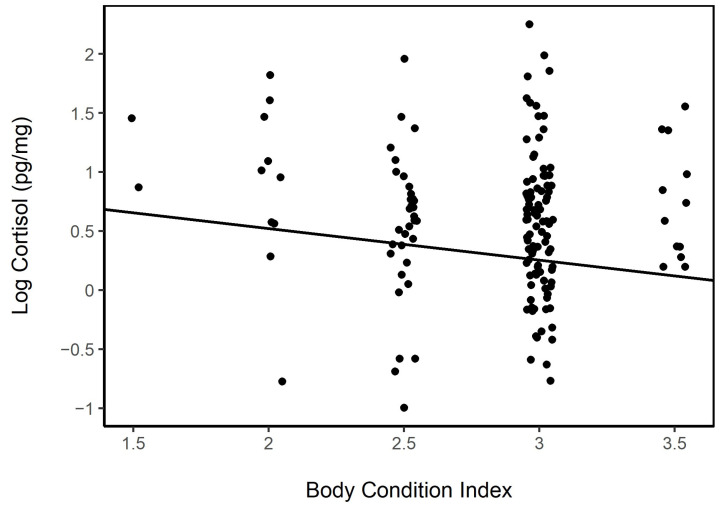
Log hair cortisol concentrations of male horses on Sable Island, NS, in relation to the body condition index (0—very poor condition, 5—obese condition). The line drawn represents the relationship between the log cortisol and body condition (LMER random factors: horse ID, age, year, and hair color; *p* = 0.028).

The BCIs among males ranged from 1.5 to 3.5, with only a few males at the extreme ends of this range (Figure 9). Age was a significant factor in the BCI among males, with 3 year olds (*n* = 30, BCI mean = 2.63; LME; fixed: age × BCI, random: horse ID, hair color, year; Intercept), 4 year olds (*n* = 15, BCI mean = 2.83; LME; *p* = 0.554), and 5 year olds (*n* = 11, BCI mean = 2.95, LME: *p* = 0.087) having lower body condition index scores than 6 year olds (*n* = 9, BCI mean = 2.94, LME: *p* < 0.001) and among adults 6 years and older (*n* = 87, BCI mean = 2.89, LME: *p* < 0.001); the BCI in this model was also significant (*p* = 0.019). 

#### 3.5.4. Social Structure and Dispersal

The harem size was significantly, and positively correlated with the HCC in males (LME; fixed: harem size, random: horse ID and hair color; *p* = 0.026), as was the band size (LME; fixed: band size, random: horse ID and hair color; *p* = 0.044), though less pronounced. 

The relationship between the HCC and whether a male was observed in a new band or new social position (e.g., Stallion to Bachelor or Bachelor to Stallion) in the year of sampling was not significant (dispersed *n* = 38, no change *n* = 114; LME; fixed: dispersed in the previous year, random: horse ID and hair color; *p* = 0.389). Likewise, the HCC in the current year was not strongly correlated to whether an individual was observed to move into a new social group or position the following year (dispersed *n* = 36, no change *n* = 116; LME; fixed: dispersed in the following year, random: horse ID and hair color; *p* = 0.167).

#### 3.5.5. Best Model of Biological and Social Variables

As completed for females, the variables found to be in the top models describing the HCC in relation to the biological and sociological factors were determined for males (See Tables S3 and S4, and Figure S2). Combining the variables from the top three biological and social variables models resulted in the following starting model, which was then evaluated for the best model fit.

(2) $$C_{iklnpvr}=B_{I}+A_{K}+Y_{L}+M_{N}+G_{P}+D_{V}+H_{R}+{\left(B\times Y\right)}_{I*L}+{\left(M\times H\right)}_{N*R}+{\left(D\times H\right)}_{V*R}+W_{E}+T_{Q}+e_{IKLNPVR}$$

where

- C = Log^10^(HCC pg·mg^−^^1^)
- Fixed effects:
  B = Body Condition Index, numerical 0–5 (I)
  A = Age, categorical with 5 levels (K = 3, 4, 5, 6, and 6+)
  Y = Year, categorical with 2 levels (L = 2011 or 2012)
  M = Bachelors in 8000 m buffer, integer (N)
  G = Bands in 8000 m buffer, integer (P)
  D = Density in 8000 m buffer, integer (V)
  H = Harem size of focal individual, integer (R)
- Random Effects:
  W = Hair color with four levels (E = black, chestnut, flaxen, and sorrel)
  T = Horse ID (Q = horse identification and character)
- $e_{IKLNPVR}$ = residual component

The variation in hair hormone concentrations in males appeared to be more directly under the biological influence of age, body condition, or related to the characteristics of the year (Table 2). Investigating the sociological variables, we observed the number of bachelors in the 8000 m buffer also appeared in some of the higher-ranked top models.

## 4. Discussion

Cortisol is a hormone regulated by a complex feedback system that is constantly responding to physiological and psychological demands, resulting in many opportunities to examine potential correlates. We focused on broad biological and social factors that occur in free-living feral horses. Importantly, variables that have not appeared significant in relation to glucocorticoid production when investigated in domestically kept horses are shown here to be significantly different when examined in the feral population of Sable Island horses. Many studies on domestically kept horses have indicated that there is no difference in HCC between males and females [38,39,40,41,90] or age [39,90]; our results differed in this respect. Frequently, these studies also include castrated males, which further reduces their comparability. We suspect that, under stabled or even among the described free-living conditions, the horses in these studies are not likely to be experiencing the full dynamic range of natural social behaviors, reproductive demands, and resource limitations to the extent which feral horses are exposed.

Along with variations in the HCC between sex and age, our key findings include that females rearing a foal had higher HCC than females that were not accompanied by a foal or yearling. For males, reproductive success is strongly linked to their social position, with most reproductive opportunities going to the dominant band stallions [65,91,92,93]; however, the HCC in males was not found to be tied to their social position as either a non-breeding bachelor or successful band stallion or tag (see, also, Medill et al. [94]). Overall, both female and male HCC were more strongly linked to biological factors such as age and body condition (and reproduction for females) or even to year effects than it was to our measures describing local population demographics. However, the higher-ranking models describing female hair cortisol levels included variables such as harem size and the number of bachelor males in the vicinity, while, for males, the number of bachelors and the overall horse density were high-ranking explanatory variables. 

Females showed a strong correlation between HCC and the presence of dependent offspring. Even females with yearlings potentially experience higher physiological demands then females without any dependent offspring based on the observed HCC. However, females accompanied by both the current year’s foal and their offspring from the previous year did not exhibit an additive increase in HCC over those with only foals present. A female is not likely to be nursing a yearling at the same time she has a new foal; however, it has been observed by this author. More likely, females were nursing the previous foal until the third trimester of their pregnancy [65]. Females often continue to nurse yearlings when either new foals are not conceived or are lost [65], which accounts for why their cortisol levels likely remain elevated compared to individuals with no dependent offspring. The cortisol concentrations in the current year were not useful to predict whether a female would successfully reproduce the following year, though a trend suggests females with lower HCC were more likely to have a foal in the following year. We compared the cortisol levels against the sex of the foal present to see if there was physiological evidence of females mobilizing resources into their raising male offspring by having higher HCC in support of the Trivers–Willard hypothesis [95,96], but this was not detected among our samples.

Body condition was a strong correlate to HCC among both males and females, with those animals in poorer conditions having higher HCC than those considered in good conditions. This was shown in both the focused models, as well as being in all the top models describing male and female HCC. Similar patterns of high HCC corresponding to poor body conditions have been observed in white-tailed deer [37]. Since cortisol is linked to the mobilization of glucose from existing stores, it is not surprising that, after a long period of high-circulating cortisol levels, individuals develop a poor body condition and have high HCC. Through the winter and into early spring, there is a period of resource limitations on Sable Island that disproportionately affects females experiencing the high-energy demands of late pregnancy [60,97]. However, during the months of June–August, vegetation is abundant on Sable Island, and nutritional needs should be met or exceeded at the time reflected in our samples. The appearance of being in a poor condition alone may not be solely related to the current energy intake; there may be other contributing factors at play keeping the HCC levels elevated. One possibility is that prolonged high levels of cortisol can also lead to impaired immune system function [1,98], which could leave individuals in this population less resistant to parasitic infections, which are known to be high [62,99]. Fecal parasite egg counts were negatively correlated with the body condition in Sable Island horses, especially among females [99], and were also higher in lactating females than in those without dependent offspring. Higher HCCs were observed in females with foals that also had the lowest BCI, reflecting the high metabolic demands of lactation.

No significant differences were found in HCC between the adult male social classes (Bachelor, Stallion, or Tag); however, Immature males (3 and 4 years old) tended to have lower HCC. Younger females also appeared to have lower HCC than the more mature 6+ females, especially among those not producing a foal. In fact, among the younger-aged females, the presence or absence of a foal resulted in a very pronounced difference in HCC. Lower cortisol concentrations among juvenile or late-pubescent horses has been detected in other populations (in serum [53]) but is not commonly seen in domestically raised horses [39,90,100]. In males of other species, this decrease in cortisol is suggested to be linked to the inhibitory influence of testosterone on the HPA axis acting prior to the onset of stress induced by reproductive competition [19,21]. 

Stability in social relationships or group hierarchies can influence cortisol production [30,101,102,103]. An increase in cortisol production has been attributed to the increase in fighting and aggression that occurs during takeovers [101]. Engh et al. [102,104] observed that instability within baboon female hierarchies led to increased cortisol, particularly amongst those females which ranks were changing. Horses that have changed social groups often find themselves facing aggression from established individuals and has been linked to increased HPA activation, as determined by serum cortisol in domestic horses [50], and among feral horses using fecal glucocorticoid metabolites [30]. The time scale that our hair samples captured was estimated to be the past 67–82 days based on the existing literature for domestic horses [70,71,72,73,74,75]. We did not observe a correlation between the observed HCC and dispersals during the period before the current survey. If changes in band memberships had occurred well outside of the time period represented by our hair samples, then individuals may have already adjusted and become established in their new hierarchy, and we are simply not detecting that dispersal event in the examined segment of hair [16,47,50]. 

Our investigation of social dispersal the year following hair cortisol sampling showed a stronger likelihood of female individuals with low cortisol levels being seen in a new social group the next year than for those which had high cortisol levels. One explanation for this could be simply that younger females (shown here to have lower HCC) are going to be more likely to leave because of natal dispersal [65,105]. Additionally, older females, particularly those with foals (both factors correlated to higher HCC), also have lower dispersal rates from their social group than subadult females [59,106]. However, the association between dispersal the following year and HCC was significant even with the inclusion of age as a random variable. This observation is counterintuitive, as being in a situation where the cortisol levels are low, suggesting the resources and social structure are adequate, should be selected for and not against. Alternatively, it could suggest that band movements are not undertaken unless the individual is in a better physiological state to take on the additional stress of establishing themselves in a new social hierarchy. The lack of a significant relationship between band changes and HCC among males is consistent with the findings of a study looking at feral horses in another population that did not detect significant changes in stallion fecal glucocorticoid levels despite inter-band movements by harem females [55]. 

The differences between the male and female top models describing HCC could suggest differences in how females and males perceive and respond to population demographics and the resulting social structures. The positive relationship between HCC and an increased harem size for males could be explained by the increased energy expended by a stallion to cover and defend a larger group. However, in the top-ranked models, it suggests that the influence of harem size on HCC is much less than the influences of age, the BCI, and year. Among females, where harem size did not have a direct effect on HCC, the inclusion of harem in the top-ranked model may be related to the influence of harem size on other factors such as reproductive success [63]. The number of bachelors present in the home range (in the second-best model) also explains some of the variations observed in female HCC. This could represent part of the physiological response leading to lower female reproductive success, which was observed in this population when the local sex ratios became more male-biased or as the band and harem sizes increased [1,60,63]. The adult sex ratio was not included as a variable in this study, as it had strong collinearity with both the number of bands and bachelors observed within the 8000 m buffer (Figures S1 and S2). However, a high number of bachelors sharing the home range (linked to high adult sex ratios) would increase the number of challenges a stallion may have to contend with, which may also influence band movement and social stability, which negatively impacts female band members [57,57,59,60]. 

One surprising observation was that females, in general, had lower HCC than males given that females were sampled at a time when many should have been experiencing greater metabolic demands related to lactation, and our sample had a higher number of lactating compared to non-lactating females. Males were also experiencing high energy requirements through mating and harem defense activities among dominant band stallions and challenging for mating opportunities by bachelors. This observation should not be concluded, as males are more “stressed” than females, but more likely, males and females potentially have different baseline levels or respond differently to physiological or psychological circumstances. Among other species, differences in HCC between male and females have been observed (vervet monkeys [21]) or found absent (grizzly bear [13], reindeer [107], and Canadian lynx [108]). Additionally, since we did the sampling at only one time of the year, we potentially missed out on variations that may be related to seasonal changes in endocrine function between the sexes [26,38,47].

While we did not find a significant year effect among the individuals sampled in both 2011 and 2012 using paired *t*-tests, we did find a significant difference testing the linear mixed effect model with the repeated samples taken into consideration. It is not altogether surprising that the paired *t*-test was not significant given that a variety of factors could have changed in different respects for different individuals, resulting in either a higher or lower HCC between the years. The observed annual difference in HCCs among all samples could potentially be explained by climatic effects and the resulting impact on resource access. Sable Island experienced lower precipitation during the summer of 2012 [57], which led to higher densities around water sources and increased the amount of time traveling between water and grazing areas. In addition to this, high overwinter mortality in 2010–2011 [57] would have potentially improved access to resources and lowered the density for the remaining individuals in the summer of 2011. This could explain why we observed the HCC in samples collected in 2012 to be higher than those collected in 2011. 

There were also variables that accounted for some of the variation in HCC that were not particularly of interest but required consideration in the model design. The most significant of these was the coarseness of the hair, which showed higher HCC levels from heavier hair (by strand segment weight). While statistically significant, the distribution of the data and low correlation coefficient indicated the influence was weak. In other studies, differences were found in the HCC between coarse guard hair and the fine undercoat from the same body location on a grizzly bear [13], and in horses, the body and mane HCC were different but remained correlated [39] and similar conclusions were made comparing horse mane and tail hairs [40]. A compounding factor that may explain some of the variations observed in this study was that hair exposed to UV may have reduced hormone concentrations [109]. Sable Island horses have no shelter from sunlight, as there are no trees and little shade on the island. The outer layers of the manes and tails of the horses are visibly faded and weathered (potentially reducing the coarseness measure), while deeper hairs retain their normal pigmentation. If our opportunistic sampling of unrestrained individuals resulted in some samples with a higher proportion of exterior (faded/weathered) hair as opposed to hairs that were better protected, this could account for some of the variability seen. In future works, the collection of deeper, more protected hairs is recommended. 

The influence of hair pigmentation on the observed cortisol concentrations has many studies supporting that HCC varies with hair color [14,32,88], while others have found no correlation to HCC [13,36] and for horses in particular [38,90]. In this study, hair color did show some indications of being an important factor to consider in the analysis. Those colors that appeared to be lower in cortisol were the lighter hairs such as flaxen (cream color) and sorrel (a mixture of greyish or light and medium brown). These phenotypes are less represented in the island’s population [76] and also in this this study, which casts some concerns as to whether the significant difference we observed is adequately supported.

One of the tenets of statistical testing is the assumption of sample independence. In our dataset, we had 17 females (15.0%) which had hair collected in both 2011 and 2012. In most of our analyses, we were able to include horse IDs as a random variable that takes this repeated sampling into consideration. One instance where this was not possible was for the GLME model to test foal presence/absence as a function of HCC. In this case, a LME model was also presented that established the significant correlation between HCC and foal presence accounting for the repeated samples. A second model where we encountered overfitting errors was investigating HCC in relation to the female body condition. The result was strongly significant (*p* < 0.001), and it was unlikely that the influence of 15% of the data having a within-subject variance unaccounted for was going to influence the conclusion when the probability was far from α = 0.05. Last, the best model describing HCC for females was created using a series of linear models without random effects added due to model overfitting errors when trying to account for the horse ID. We are still relatively confident that the top-ranked models represented the strongest and most influential variables. During data exploration, the removal of a lower-ranked fixed variable allowed the inclusion of the horse ID as a random factor, and the resulting top models still included the same variables with similar estimates (see the Supplemental Materials). Still, we acknowledge that we broke the assumption of independence in a handful of tests and encourage our readers to weigh that when reviewing the results. 

## 5. Conclusions

The social and physiological data of the long-term individual-based research on the Sable Island horses has allowed for detailed investigations of hair cortisol correlates and patterns in a feral population. Both male and female HCC are linked more strongly to physiological inputs such as body condition, age, year effects, and, for females, the presence of dependent offspring. The data suggest that the male and female HPA axes may respond differently to other cues relating to social structures. In the top-ranked models, females had more variations in HCC explained by the factors of harem size and bachelor abundance. This information could suggest the physiological link to what we have learned about the female fitness response to changes in band membership and dispersal [59,63] or population demographics [57,61]. Our research also suggests that HCC patterns observed in feral horse populations can be quite different from those observed in domestically kept horses, likely due to experiencing resource limitations, reproductive demands, parasite loads, and the ability to express all natural behaviors.

## Figures and Tables

**Table 1:** Modeling of female log hair cortisol concentrations (HCC) to the top social and biological factors. Variables include age, abundance of bachelors (Bach), number of other bands, and density of horses within the home range, along with the body condition index (BCI), presence or absence of a foal (Foal), harem size (Harem), and year. The model also included the top interaction terms from previous models: age × foal, age × BCI, and bachelors × harem size. An asterisk (*) indicates the inclusion of factor variables in the model. Only variables observed in the top models are reported. No random factors are included due to model overfitting. Null model AIC_c_ = 180.09.

Intercept	Age	BCI	Harem	Age * BCI	Bach	Foal	Foal * Age	Bach * Harem	Year	df	AIC_c_	∆AIC_c_	logLik
2.635	*	−0.865	−0.041	*						12	138.4	0.00	−55.89
2.249	*	−0.817	−0.047	*	0.016					13	138.5	0.06	−54.68
0.202	*	−0.376	0.233		0.059	*	*	−0.014		15	138.9	0.42	−52.32
1.600	*	−0.769	0.140	*	0.045			−0.010		14	139.4	0.97	−53.88
2.497	*	−0.837	−0.044	*					*	13	139.5	1.07	−55.18
2.590	*	−0.895		*						11	139.7	1.29	−57.75
1.008	*	−0.396	−0.040		0.017	*	*			14	139.9	1.41	−54.10
2.455	*	−0.809	−0.039	*		*				13	140.1	1.62	−55.46
3.015	*	−1.031		*		*	*			16	140.1	1.68	−51.65
1.295	*	−0.403	−0.034			*	*			13	140.2	1.76	−55.53
3.056	*	−1.009	−0.033	*		*	*			17	140.2	1.76	−50.37
1.185	*	−0.402				*	*			12	140.3	1.82	−56.79
2.707	*	−0.972	−0.039	*	0.015	*	*			18	140.4	2.01	−49.14

**Table 2:** Linear mixed effect models using a combination of biological and social explanatory variables to explain the log hair cortisol concentrations in male feral horses. The included fixed factors are age, body condition index (BCI), year, number of bachelors (Bach), and bands in an 8000 m radius (Bands), horse density in the 8000 m radius, and the interaction between the year and BCI (BCI × Year) and between the number of bands and horse density (Bands × Density). An asterisk (*) indicates inclusion of a factor variable in the model. Factors not in the top models are not shown in the table. Random terms: horse ID and hair color, null model AIC_c_ = 283.6.

Intercept	Age	BCI	Year	Density	Bands	Bach	BCI × Year	df	AIC_c_	∆AIC_c_	logLik
−0.501	*	0.093	*				*	11	247.4	0.00	−111.78
0.592	*	−0.277	*					10	247.5	0.05	−112.97
0.370	*	−0.271	*			0.017		11	248.6	1.16	−112.36
0.257	*	−0.280				0.034		10	248.8	1.31	−113.60
−0.651	*	0.081	*			0.015	*	12	248.8	1.34	−111.27
−0.498	*	0.120	*	−0.003			*	12	249.1	1.65	−111.43
0.612	*	−0.257	*	−0.003				11	249.2	1.79	−112.68
−0.337	*	0.068	*		−0.003		*	12	249.4	1.94	−111.57
0.521	*	−0.334			−0.008	0.041		11	249.4	1.95	−112.76
0.762	*	−0.303	*		−0.004			11	249.4	1.96	−112.76
0.603	*	−0.024	*		−0.007	0.024		12	249.6	2.12	−111.66

## Data Availability

The data presented in this study are available on request from the corresponding author.

## Supplementary Materials

The following supporting information can be downloaded at: https://www.mdpi.com/article/10.3390/ani13132133/s1: Equation (S1); Table S1: Modeling of log hair cortisol concentrations to biological factors in females. The top models include the body condition index (BCI), Age, presence or absence of a foal (Foal), and Year, as well as the interaction terms Age × Foal and Age × BCI. An asterisk indicates inclusion of a factor variable in the model. No random factors were included due to a singularity error. Variables not included in the resulting top models are not shown. Null model AIC_c_ = 180.09.; Figure S1: Investigation of the social variables for collinearity among the female dataset (R^2^ score indicated in the matrix, Zuur et al. 2010), median (longitude position on the island), adult sex ratio (ASR), and band size were dropped from further analysis, and Bachelors (Bach), Bands, Density, and Harem size were retained.; Equation (S2); Table S2: Linear mixed effect models of log hair cortisol concentrations to sociological factors in female horses of Sable Island. Variables include the abundance of Bachelors in 8000 m (Bach), number of bands in the 8000 m buffer (Band), horse density in the vegetated portion of the 8000 m buffer, and number of females ≥2 years old in the focal individual’s band (Harem), as well as all two-way interaction terms between these variables. Only variables found in the top models are shown. No random variables included due to singularity errors (model overfitting). Null model AIC_c_ = 180.09.; Equation (S3); Table S3: Modeling of log hair cortisol concentrations to biological factors in male horses of Sable Island, NS. Fixed variables include age, body condition index (BCI), social position, median location (Longitude), and year, and all two-way interactions terms were included. An asterisk indicates inclusion of a factor variable in the model. Random factors: horse ID and hair color. Only variables present in the top models are shown. Null model AIC_c_ = 283.6.; Figure S2: Investigation of social variables for collinearity among the male dataset (R^2^ score indicated in matrix, Zuur et al. 2010), Median (longitudinal position on the island), adult sex ratio (ASR), and band size were dropped from further analysis, and Bachelors (Bach), Bands, Density, and Harem size retained.; Equation (S4); Table S4: Linear mixed effect modeling of log hair cortisol concentrations to sociological factors in male horses of Sable Island, NS. Variables include an abundance of Bachelors (Bach), number of bands (Bands), and horse density (Density) all measured within 8000 m of individual median locations and the number of females ≥2 years old in the focal individual’s band (Harem), as well as the two-way interaction terms between these variables. Random effects included in the model: horse ID and hair color. Null model AIC_c_ = 283.6.
