# Supplementary material for: Hair Cortisol Concentrations in Feral Horses and the Influence of Physiological and Social Factors
Source: Animals (Basel). 2023 Jun 27;13(13):2133. doi: 10.3390/ani13132133 (PMC10339936; doi:10.3390/ani13132133)
Supplement: Supplementary file 1 [file animals-13-02133-s001.zip › animals-2422215-supplementary.pdf]

Supplemental Material

Biological Model Describing Female HCC

Equation S1:

$$C_{ijklz} = B_I + F_J + A_K + Y_L + X_Z + (B \times F)_{I*J} + (B \ \times A)_{I*K} + (B \ \times Y)_{I*L} + (F \ \times A)_{J*K} + (F \ \times Y)_{J*L} + e_{IJKL}$$

Where:

- C = Log^{10}(HCC)
- Fixed Effects:
  - B – Body condition index, numerical 0-5 (I)
  - F – Foal with 2 levels (J = Presence or Absence)
  - A – Age, categorical with 5 levels (K = 3, 4, 5, 6, and 6+)
  - Y – Year, categorical with 2 levels (L = 2011 or 2012)
  - X – Median Longitude, numerical (Z)
- e_{IJKLZ} – residual component

Due to uneven sample sizes and few levels of some factors, attempting to add random variable horse ID led to model overfitting. We did evaluate dropping Year and was then able to include Horse ID as random variable. The resulting top two models did not change with regards to what variables were included and minimal change in estimate values were observed.

Table S1. Modeling of log hair cortisol concentration to biological factors in females. Top models include body condition index (BCI), Age, presence or absence of a foal (Foal), Year, as well as the interaction term Age×Foal and Age×BCI. An asterisk indicates inclusion of factor variable in the model. No random factors included due to singularity error. Variables not included in the resulting top models are not shown. Null model AIC_c = 180.09.

Intercept	BCI	Age	Foal	Year	Age×BCI	Age×Foal	df	AIC_c	ΔAIC_c	logLik
**2.590**	-0.895	*			*		11	136.3	0.00	-56.02
**1.177**	-0.399	*	*			*	12	137.6	1.33	-55.47
**2.467**	-0.872	*		*	*		12	137.7	1.35	-55.48
**3.015**	-1.031	*	*		*	*	16	137.9	1.56	-50.51
**2.416**	-0.417	*	*		*		12	137.9	1.62	-55.62
**1.268**	-0.392	*	*				8	138.2	1.86	-60.48
**1.084**	-0.409	*	*	*		*	13	138.6	2.29	-54.71

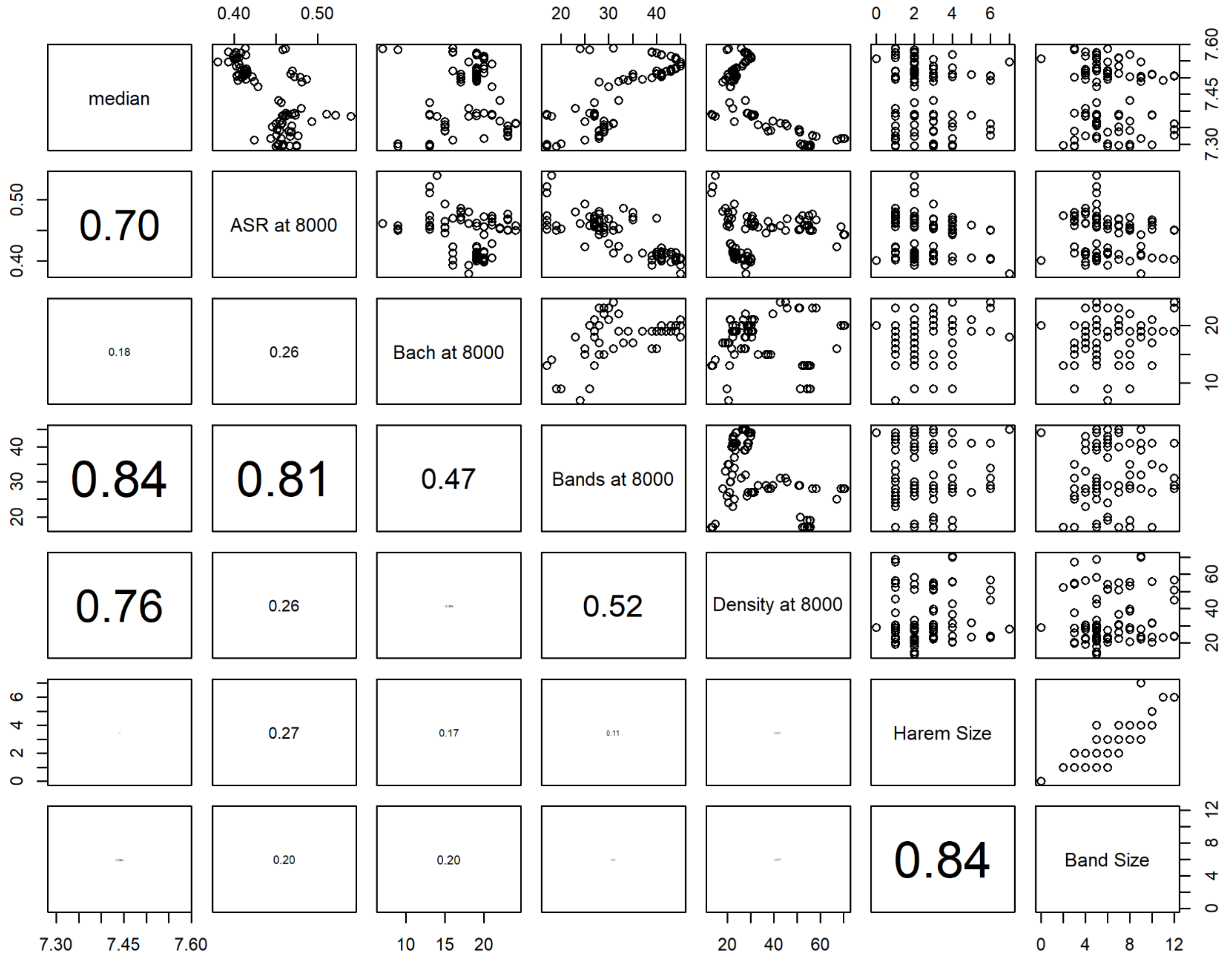

Figure S1: Investigation of social variables for collinearity among the female dataset (R^2 score indicated in matrix, Zuur et al. 2010), median (longitude position on island), adult sex ratio (ASR), and Band Size were dropped from further analysis and Bachelors (Bach), Bands, Density and Harem size were retained.

Social factors model for female HCC:

Equation S2

$$C_{npvr} = M_N + G_P + D_V + H_R + (M \times G)_{N*P} + (M \times D)_{N*V} + (M \times H)_{N*R} + (G \times D)_{P*V} + (G \times H)_{P*R} + (D \times H)_{V*R} + e_{NPVR}$$

Where:

- C – Log^{10}(HCC pg/mg)
- Fixed effects:
  - M – Bachelors in 8000m buffer, integer (N)
  - G – Bands in 8000m buffer, integer (P)
  - D – Density in 8000m buffer, integer (V)
  - H – Harem size of focal individual, integer (R)
- e_{NPVR} – residual component

Table S2. Linear mixed effect models of log hair cortisol concentration to sociological factors in female horses of Sable Island. Variables include abundance of bachelors in 8000 m (Bach), number of bands in 8000 m buffer (Band), horse density in the vegetated portion of 8000m buffer, and number of females ≥ 2 years old in the focal individual's band (Harem), as well as all two-way interaction terms between these variables. Only variables found in the top models are shown. No random variables included due to singularity errors (model overfitting). Null model AIC_c = 180.09.

Intercept	Bach	Band	Harem	Density	Bach× Harem	Bach× Density	Harem× Density	df	AIC_c	ΔAIC_c	logLik
**-1.399**	0.105		0.452		-0.027			5	169.6	0.00	-79.54
**-1.386**	0.100	0.002	0.438		-0.026			6	171.6	2.02	-79.45
**-1.425**	0.105		0.451	0.001	-0.027			6	171.7	2.14	-79.51
**-1.901**	0.133		0.328	0.020	-0.020	-0.001		7	172.4	2.80	-78.72
**-1.493**	0.097		0.469	0.008	-0.023		-0.003	7	172.5	2.89	-78.76

Biological Model Describing Male HCC

Equation S3:

$$C_{iwklz} = B_I + S_W + A_K + Y_L + X_Z + (B \times S)_{I*W} + (B \times A)_{I*K} + (B \times Y)_{I*L} + (B \times X)_{I*Z} + (S \times A)_{W*K} + (S \times Y)_{W*L} + (S \times X)_{W*Z} + (A \times Y)_{K*L} + (A \times X)_{K*Z} + (Y \times X)_{L*Z} + W_E + T_Q + e_{IWKL}$$

Where:

- C – Log^{10}(HCC)
- Fixed Effects:
  - B – Body condition index, numerical 0-5 (I)
  - S – Social Position with 4 levels (W = Bachelor, Immature, Stallion, Tag)
  - A – Age, categorical with 5 levels (K = 3, 4, 5, 6, and 6+)
  - Y – Year, categorical with 2 levels (L = 2011 or 2012)
  - X – Median Longitude, numerical (Z)
- Random Effects:
  - W – Hair color with 4 levels (E = Black, Chestnut, Flaxen, Sorrel)
  - T – Horse ID (Q = horse identification, character)
- e_{IJKLZ} – residual component

Table S3. Modeling of log hair cortisol concentration to biological factors in male horses of Sable Island, NS. Fixed variables include age, body condition index (BCI), social position, median location (Longitude), year, and all two-way interactions terms were included. An asterisk indicates inclusion of factor variable in model. Random factors: horse ID and hair color. Only variables present in the top models are shown. Null model AIC_c = 283.6.

Intercept	Age	Year	BCI	Longitude	BCI×Year	df	AIC_c	ΔAIC_c	logLik
**-0.501**	*	*	0.093		*	11	247.4	0.00	-111.78
**0.592**	*	*	-0.277			10	247.5	0.05	-112.97
**3.464**	*	*	-0.304	-0.377		11	249.3	1.84	-112.70
**1.801**	*	*	0.059	-0.297	*	12	249.5	2.03	-111.62
**-0.159**	*	*				9	250.5	3.08	-115.63

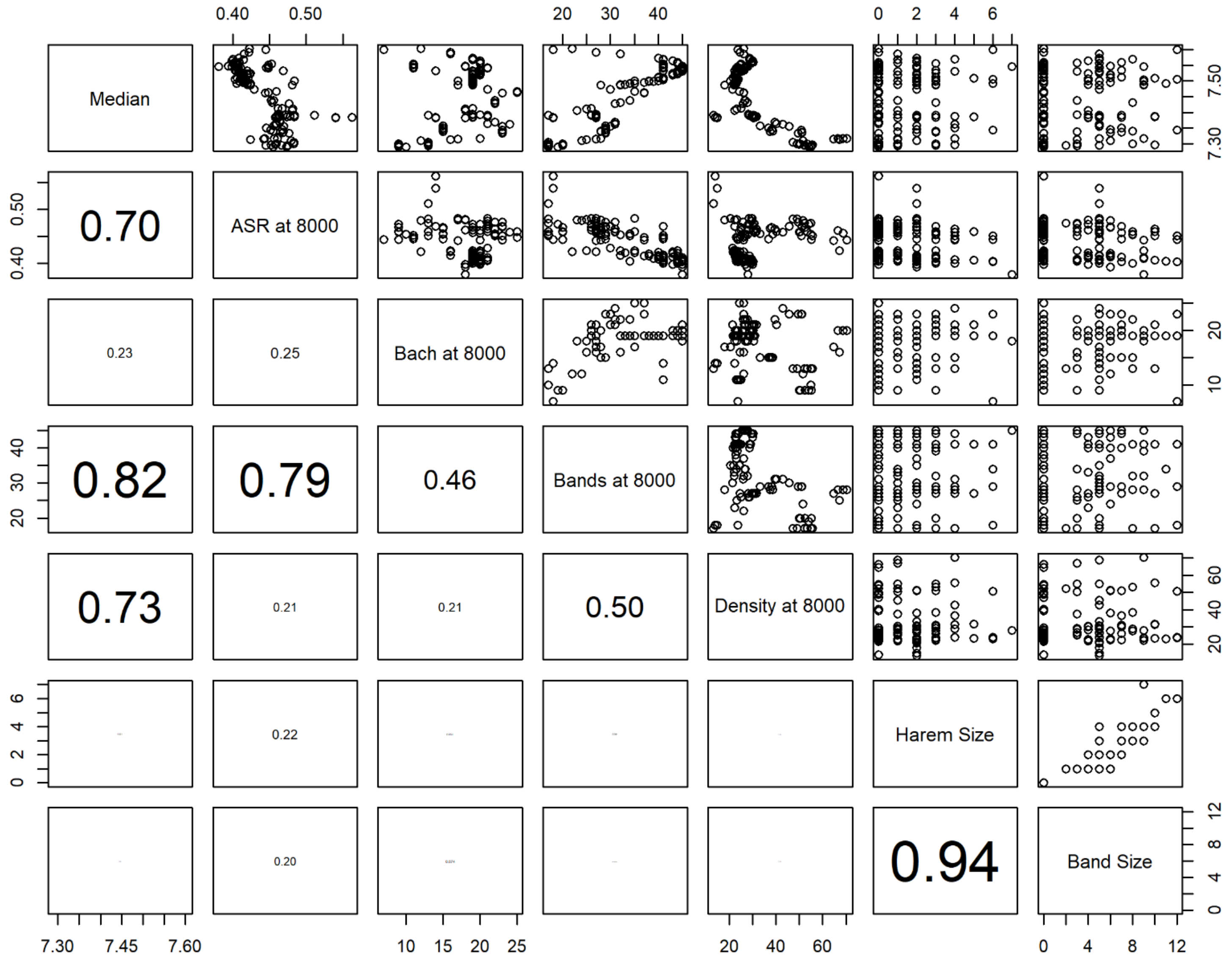

Figure S2: Investigation of social variables for collinearity among the male dataset (R^2 score indicated in matrix, Zuur et al. 2010), Median (longitudinal position on island), Adult Sex Ratio (ASR), and Band Size were dropped from further analysis and Bachelors (Bach), Bands, Density and Harem size retained.

Social factors model for male HCC

Equation S4:

$$C_{npvr} = M_N + G_P + D_V + H_R + (M \times G)_{N*P} + (M \times D)_{N*V} + (M \times H)_{N*R} + (G \times D)_{P*V} + (G \times H)_{P*R} + (D \times H)_{V*R} + W_E + T_Q + e_{NPVR}$$

Where:

- C – Log^{10}(HCC pg/mg)
- Fixed effects:
  - M – Bachelors in 8000m buffer, integer (N)
  - G – Bands in 8000m buffer, integer (P)
  - D – Density in 8000m buffer, integer (V)
  - H – Harem size of focal individual, integer (R)
- Random Effects:
  - W – Hair Color with 4 levels (E = Black, Chestnut, Flaxen, Sorrel)
  - T – Horse ID (Q = horse identification, character)
- e_{NPVR} – residual component

Table S4: Linear mixed effect modeling of log hair cortisol concentration to sociological factors in male horses of Sable Island, N.S. Variables include abundance of bachelors (Bach), number of bands (Bands), horse density (Density) all measured within 8000m of individuals median location, number of females ≥ 2 years old in the focal individual's band (Harem) as well as the two-way interaction terms between these variables. Random effects included in model: horse ID and hair color; Null model AIC_c = 283.6.

Intercept	Bach	Harem	Band	Density	Bach* Harem	Harem* Density	Band* Density	df	AIC_c	ΔAIC_c	logLik
**-0.548**	0.051	0.254			-0.011			7	273.8	0.00	-129.51
**-0.272**	0.035	0.055						6	274.5	0.67	-130.94
**-0.478**	0.057	0.258	-0.007		-0.011			8	274.9	1.10	-128.95
**-0.858**	0.055	0.381		0.008	-0.011	-0.004		9	275.2	1.37	-127.95
**-1.715**	0.070	0.283	0.033	0.038	-0.012		-0.001	10	275.2	1.43	-126.84
**-1.774**	0.072	0.383	0.028	0.039	-0.012	-0.003	-0.001	11	275.4	1.60	-125.76
**-0.200**	0.041	0.056	-0.006					7	275.7	1.87	-130.44
**-0.640**	0.052	0.263		0.002	-0.011			8	275.7	1.89	-129.34
**-0.541**	0.038	0.174		0.007		-0.004		8	276.0	2.23	-129.51
